# Case Report: ^18^F-PSMA PET/CT May Improve the Clinical Management of Penile Metastases From Prostate Cancer

**DOI:** 10.3389/fonc.2021.683343

**Published:** 2021-05-13

**Authors:** Junjie Fan, Hua Liang, Xing Zhang, Xingfa Chen, Xiaoyi Duan, Lei Li, Dalin He, Kaijie Wu

**Affiliations:** ^1^ Department of Urology, First Affiliated Hospital of Xi’an Jiaotong University, Xi’an, China; ^2^ Department of Urology, Baoji Central Hospital, Baoji, China; ^3^ Department of Pathology, First Affiliated Hospital of Xi’an Jiaotong University, Xi’an, China; ^4^ Department of Radiology, First Affiliated Hospital of Xi’an Jiaotong University, Xi’an, China

**Keywords:** PSMA PET/CT, prostate cancer, penile metastases, early diagnosis, survival advantage

## Abstract

Metastases from prostate cancer (PCa) to the penis are extremely rare, and few case reports exist in the literature. Because most patients usually present with multiple distant metastases at diagnosis, the prognosis is very poor. With the wide application of prostate-specific membrane antigen (PSMA) PET/CT, penile metastases may be detected at an early stage. Thus, questions regarding whether early diagnosis and precise treatment will equate to a survival advantage have recently been raised. In the present study, we reported 3 cases of penile metastasis from castration-resistant PCa. Moreover, a patient with asymptomatic penile metastases was diagnosed by ^18^F-PSMA PET/CT followed by lesion biopsy, and the prognosis was very well, despite with an aggressive pathological feature and low treatment intensity. In addition, we performed a literature review and found 62.5% of asymptomatic penile metastases were diagnosed by PSMA PET/CT in past seven years. Thus, we believe that PSMA PET/CT may detect more asymptomatic penile metastases in future, which led to early diagnosis, treatment and survival advantage.

## Introduction

The common sites of metastasis from prostate cancer (PCa), in order of decreasing frequency, are the bone, pelvic lymph nodes, lung and liver. Metastases from PCa to the penis are extremely rare, with a frequency of less than 0.3% (1, 2). The prognosis for these patients is very poor, as they usually present with disseminated disease. Kotake et al. reviewed the data of 25 PCa patients with penile metastasis and found that 41% of patients died within 6 months after diagnosis (1). However, the incidence of penile metastasis from PCa may be more frequent than previously thought, with wide application of prostate-specific membrane antigen (PSMA) PET/CT. In addition, penile metastasis from PCa may be detected at an early stage. Thus, questions regarding whether accurate diagnosis and appropriate treatment lead to better outcomes have recently been raised.

## Case Description

### Case 1

A 77-year-old male presented to our clinic in October 2009 with a 5-year history of dysuria. The serum prostate-specific antigen (PSA) level was 1145 ng/ml, and prostate biopsy was subsequently performed. The histopathology showed prostatic adenocarcinoma (Gleason score: 5 + 5 = 10). No bone metastases were observed by bone scan. Then, the patient received hormonal therapy (bilateral orchiectomy and 50 mg/day bicalutamide).

**Figure 1:**
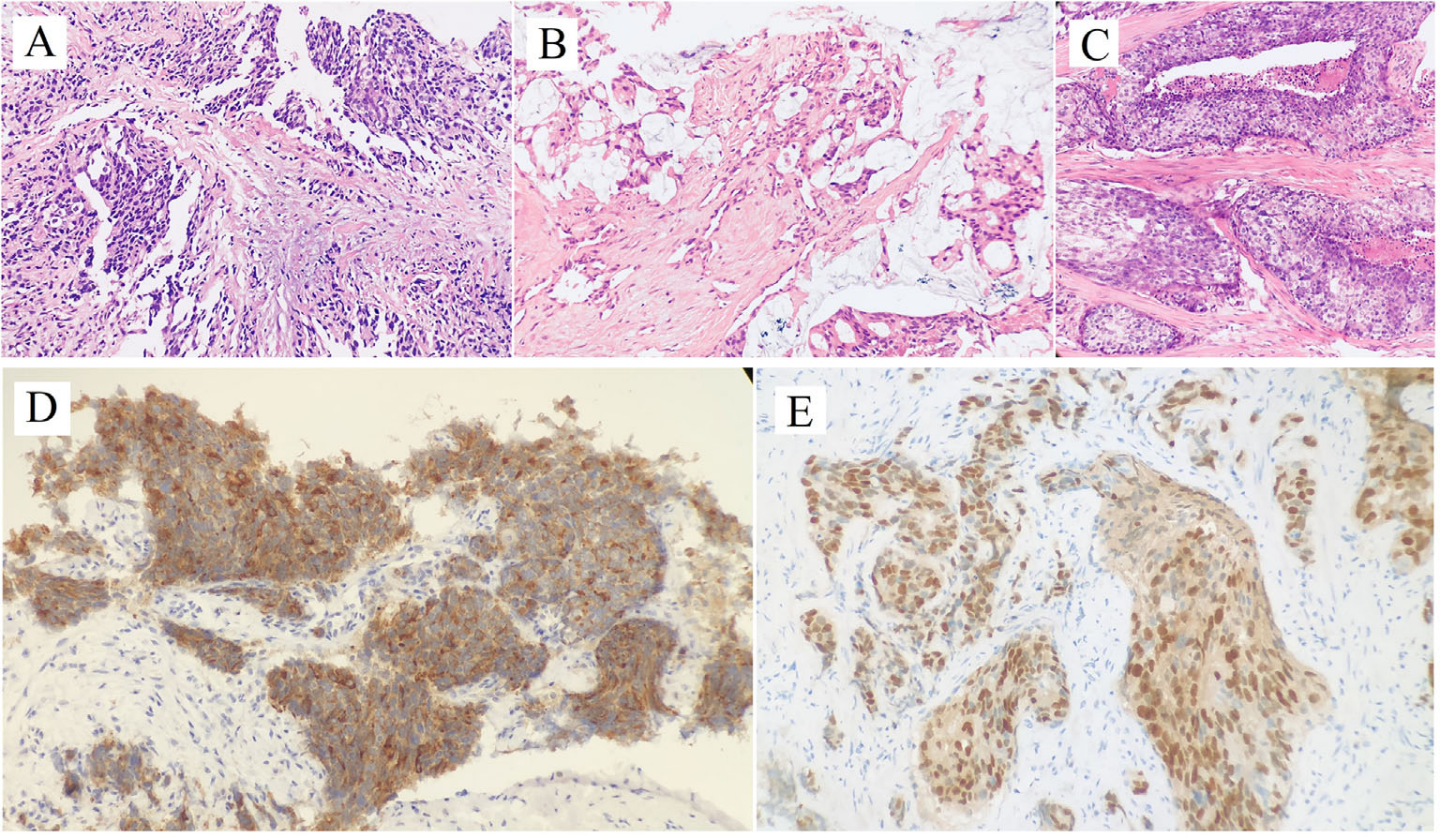
Hematoxylin and Eosin staining of penile metastatic lesions showed poorly differentiated prostatic adenocarcinoma (**A**: case 1; **B**: case 2; **C**: case 3). Two micrographs illustrate **(D)** PSA+, **(E)** NKX3.1+ tumor cells of the penile metastatic lesion in case 3.

Regular follow-up showed no evidence of tumor recurrence until November 2010. He presented with a 2-month history of priapism, and the serum PSA level was found to be 0.09 ng/ml. Doppler ultrasonography revealed a high-resistance flow pattern in the penile artery. No lymph nodes and bone metastases were observed. Then, radical penectomy was performed, and the histopathologic examination of the surgical specimen revealed diffuse, poorly differentiated adenocarcinoma cells growing in the corpora cavernosa (**Figure 1A**). An immunostaining panel revealed that the tumor cells were positive for prostatic acid phosphatase but negative for PSA, which was consistent with metastatic prostatic adenocarcinoma. The patient refused radiotherapy and chemotherapy but continued hormonal therapy. After one year, multiple, subcutaneous, nontender, erythematous nodules over the right lower leg were found, and the PSA level increased to 0.26 ng/ml. Biopsy of a skin nodule revealed neoplastic cells with prominent atypical nucleoli, brisk mitotic activity, and clear cytoplasm, consistent with metastatic adenocarcinoma, and tumor cells were positive for prostatic acid phosphatase. These findings confirmed the diagnosis of metastatic PCa to the skin. The patient refused further active treatment and died due to progression of the disease 3 months after initial presentation with skin metastases. A timeline with relevant data of Case 1 was shown in **Figure 3**.

### Case 2

A 76-year-old male presented to our clinic in November 2019 with a 2-month history of a painless lump at the base of the penis. Four years previously, he was diagnosed with metastatic prostatic adenocarcinoma based on a markedly elevated serum PSA level (2241 ng/ml), a left supraclavicular mass, imaging studies, and pathologic results of ultrasound-guided core needle biopsy of the supraclavicular mass and prostate. The histopathology results showed prostate adenocarcinoma (Gleason score: 4 + 4 = 8). No bone metastases were observed by bone scan. Then, he received intermittent hormonal therapy (1.2 mg/month goserelin and 50 mg/day bicalutamide).

After hormonal therapy, the left supraclavicular mass gradually disappeared, and the PSA level decreased to 0.01 ng/ml (October 2015). In November 2019, the patient found a painless lump at the base of the penis, and the serum PSA level was up to 21.4 ng/ml. Magnetic resonance imaging (MRI) showed prostate cancer invading the bladder neck and a heterogeneous mass of the bulb of the penis. Moreover, multiple bone metastases were observed by bone scan. Then, ultrasound-guided biopsy of the penile lump was performed, and the histopathology results of the specimen showed poorly differentiated prostatic adenocarcinoma (**Figure 1B**). Because of personal preference, the patient chose to receive abiraterone acetate (1000 mg/day) plus prednisone (10 mg/day). After treatment, the mass on the penis gradually disappeared, and serum PSA decreased to 7.6 ng/ml (September 2020). A timeline with relevant data of Case 2 was shown in **Figure 3**.

### Case 3

A 72-year-old male who was presented in March 2017 with a 13-year history of worsening dysuria. The volume of his prostate was measured at 70 mL by transrectal ultrasound, and the PSA level was normal. Urinary flow rate assessment showed a Qmax was only 6 mL/s, and postvoid residual urine was 220 mL. Then he was diagnosed as benign prostatic hyperplasia and the transurethral resection of the prostate was performed. The histopathology examination of the surgical specimen showed poorly differentiated prostatic adenocarcinoma, and the serum PSA level after the operation was 1.3 ng/ml. Because of personal preference and economic burden, the patient chose to receive oral bicalutamide (50 mg/day) and was maintained under surveillance for serum PSA every 3 months.

In April 2018, the serum PSA level was found to be 2.64 ng/ml, and multiparametric MRI showed a heterogeneous mass in the prostate, extensively invading the bladder neck, bilateral seminal vesicles and anterior rectum wall. No bone or pelvic lymph node metastases were observed. Then, he received transrectal ultrasound-guided prostate biopsy, and histopathology verified prostate adenocarcinoma with focal intraductal carcinoma (Gleason score: 4 + 4 = 8). An immunostaining panel revealed that the tumor cells were positive for HCK, P63, CK5/6 and P53 but were negative for Syn and CD56. Subsequently, the patient underwent radical radiotherapy and hormonal therapy (1.2 mg/month goserelin and 50 mg/day bicalutamide).

**Figure 2:**
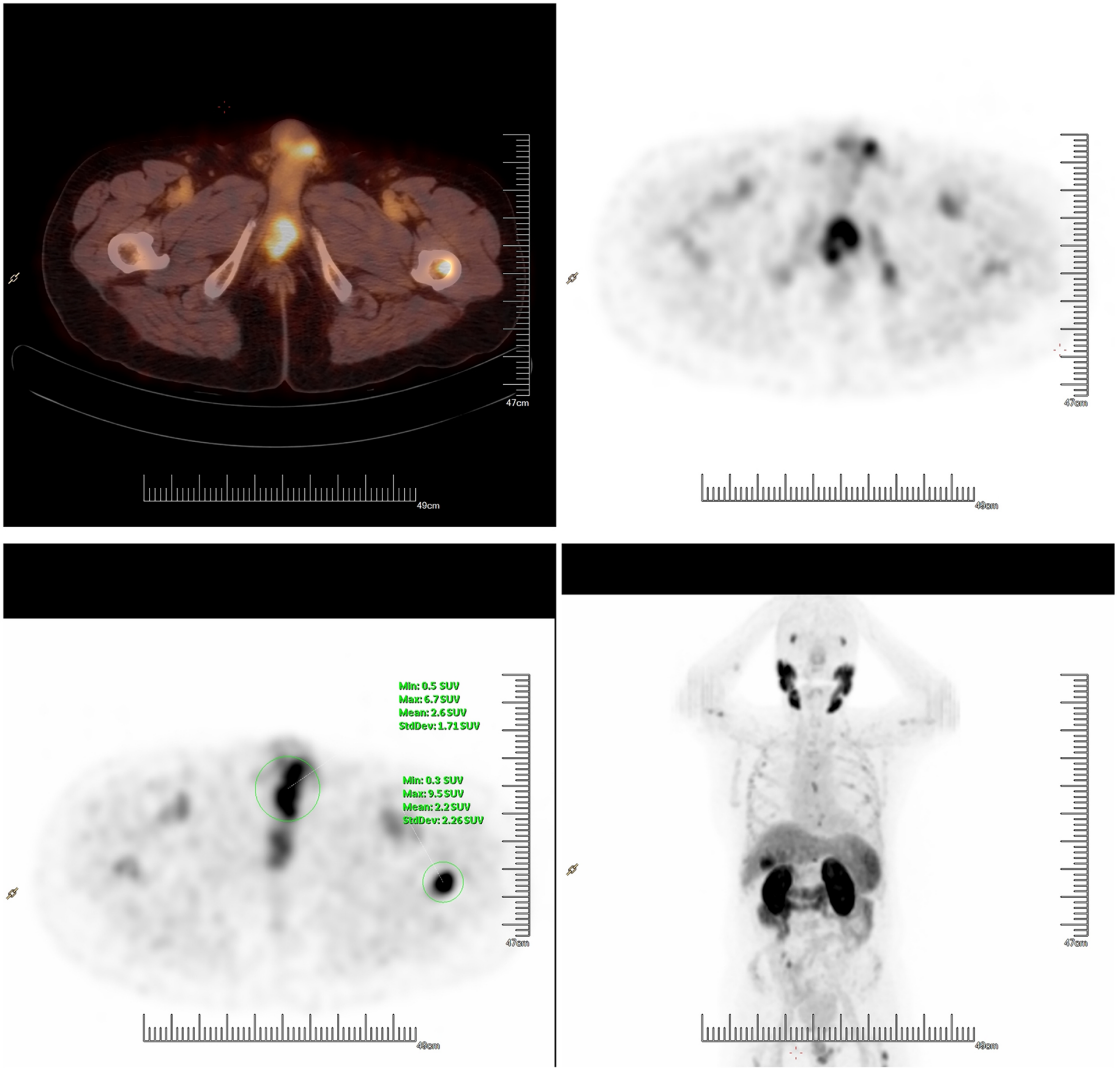
^18^F prostate-specific membrane antigen PET/CT showed suspicious tracer uptake in the base of the penis and multiple bone metastases.

**Figure 3:**
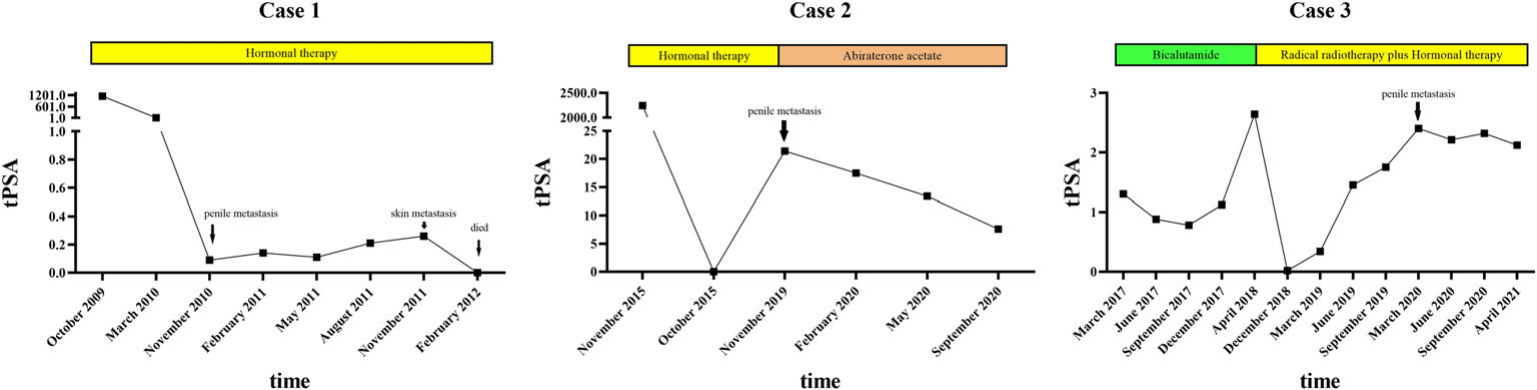
A timeline with relevant data of the 3 cases.

After radiotherapy, the PSA level decreased to 0.018 mg/L (December 2018). The patient remained asymptomatic until March 2020, and serum PSA was found to be 2.4 ng/ml. ^18^F-prostate specific membrane antigen (^18^F-PSMA) PET/CT was performed and revealed suspicious tracer uptake in the base of the penis as well as multiple bone metastases (**Figure 2**). Ultrasound-guided core needle biopsy was performed, and the histopathology results showed poorly differentiated prostatic adenocarcinoma (Gleason score: 5 + 5 = 10) (**Figure 1C**). An immunostaining panel revealed that the tumor cells were positive for PSA (**Figure 1D**) and NKX3.1 (**Figure 1E**). The patient refused radical penectomy and chemotherapy while opting for continuing hormonal therapy (1.2 mg/month goserelin and 50 mg/day bicalutamide). Until now, there has been no evidence of tumor recurrence or progression, and the recent serum PSA level was 2.12 ng/ml (April 2021). A timeline with relevant data of Case 3 was shown in **Figure 3**.

## Discussion

Although the corpora cavernosum of the penis is rich in blood supply, the incidence of penile metastases from PCa is extremely low. A plausible explanation for its presumed low incidence may be that imaging for PCa does not typically include the penis. PSMA is a type II transmembrane protein that is overexpressed on the cell membrane of nearly all prostatic cancer cells, especially in advanced-stage and castration-resistant PCa (3). Thus, PSMA is a promising and specific target for PCa imaging and shows great promise for improving the management of patients with PCa.

Comparing conversation imaging (i.e., bone scan, CT and MRI), PSMA PET/CT does not increase the accuracy of primary staging but identifies distant uncommon site metastases, such as those in the brain and penis. To provide a summary list of case reports, we performed a search of PubMed-indexed biomedical journals. We identified 17 individual cases with penile metastases from PCa reported between 2015 and January 2021, and PET/CT had gradually become the main imaging diagnoses of penile metastases (**Table 1**). Of 8 cases with asymptomatic penile metastases, 5 cases (62.5%) were diagnosed by PSMA PET/CT, especially for ^68^Ga-PSMA PET/CT. Vadi et al. reported that a patient with PCa was diagnosed with penile metastases by ^68^Ga-PSMA PET/CT and underwent ^177^Lu-labeled PSMA radioligand therapy (12). However, patient clinical course information or patient outcomes were not detailed. Recently, Mansbridge et al. outlined a history of castration-resistant PCa patients with asymptomatic penile metastases found on PSMA PET/CT (15). Currently, ^68^Ga-PSMA is a widely used tracer for PET imaging applications in the detection and staging of PCa. However, the disadvantage of ^68^Ga-PSMA PET/CT is that it has more bladder activity, which may influence the uptake evaluation of the prostate bed. ^18^F-PSMA is a novel PSMA-based radiopharmaceutical that has several advantages over ^68^Ga-PSMA, such as high labeling yields, outstanding tumor uptake and fast, nonurinary background clearance. Hence, it may have better diagnostic performance than ^68^Ga-PSMA in local recurrence and micrometastases. To our knowledge, this is the first report of asymptomatic penile metastases detected by ^18^F-PSMA PET/CT. In our report, ^18^F-PSMA PET/CT revealed unusual tracer uptake in the base of the penis, and the lesion was subsequently proven to be metastatic adenocarcinoma from the prostate by biopsy. Thus, we assumed that PSMA PET/CT, especially using ^18^F-labeled tracers, may lead to more reports of incidental cases in the future and broaden our understanding of the pathogenesis of penile metastases.

**Table 1:** Reported Cases of Penile Metastases from Prostate Cancer in the Past Few Years.

Investigation	Years	Age	GS of the primary tumor lesion	Initially local Symptoms	PSA (ng/ml)	Imaging	Location	Treatment	Survival (Month)
Gaspar (4)	2015	52	5+3 = 8	a priapism and nodules	4.56	CT	corpora cavernosa	ADT (bicalutamide)	2
Soma (5)	2015	74	N.A.	multiple non tender nodules	227	CT	N.A.	palliative therapy	4
Roma (6)	2015	76	3+5 = 8	urinary hesitancy, pain and urinary urgency	<0.05	None	penile urethra	cystectomy, urethrectomy and penile resection	N.A.
Fiaschetti (7)	2016	84	5+4 = 9	None	8.81	CT and MRI	corpora cavernosa	estramustine phosphate	30
Luca (8)	2016	62	4+5 = 9	persistent and painful erection	N.A.	MRI	corpora cavernosa	palliative radiotherapy, a pudendal nerve block and ADT	N.A.
Atag (9)	2017	84	3+3 = 6	painless reddish solitary nodules	4.57	^18^F FDG PET/CT	penile skin	chemotherapy (cabazitaxel)	15
Dureja (10)	2017	59	4+4 = 8	None	9.8	^68^Ga PSMA PET/CT	Penile shaft and the corpora cavernosa	N.A.	N.A.
Kamaleshwaran (11)	2018	79	3+4 = 7	painful urinary outflow obstruction and persistent erection.	>100	^68^Ga PSMA PET/CT	penile shaft	ADT (bilateral orchidectomy) and radiotherapy	N.A.
Vadi (12)	2018	85	5+4 = 9	pain in multiple skeletal sites	78	^68^Ga PSMA PET/CT	penile shaft	Lu177 PSMA radioligand therapy	1
Wong (13)	2019	83	4+5 = 9	a painless lump	N.A.	MRI	corpora cavernosa	ADT	N.A.
Salavati (14)	2020	85	4+4 = 8	None	1	^18^F-fluciclovine PET/CT	penile shaft	external beam radiation therapy	12
Mansbridge (15)	2020	69	4+5 = 9	None	83	PSMA PET/CT	N.A.	ADT (Enzalutamide) and palliative radiotherapy (30 Gy/10#)	18
Tatkovic (16)	2020	71	N.A.	None	110	^68^Ga PSMA PET/CT	penile shaft	Lu177 PSMA radioligand therapy	N.A.
		72	N.A.	None	0.74	^68^Ga PSMA PET/CT	penile shaft	ADT	N.A.
		80	N.A.	None	69	^68^Ga PSMA PET/CT	penile shaft	ADT	N.A.
		85	N.A.	N.A.	27	^68^Ga PSMA PET/CT	penile shaft	N.A.	N.A.
Bianchi (17)	2021	81	4+3 = 7	None	7.3	^18^F-FCH PET/CT	corpora cavernosa	palliative therapy	8

PSA, Prostate-Specific Antigen; CT, Computed Tomography; MRI, Magnetic Resonance Imaging; PSMA, Prostate-Specific Membrane Antigen; FCH, fluorocholine; GS, Gleason Score; N.A., not available.

The possible mechanisms of penile metastasis from PCa have been explained as follows: 1) direct invasion; 2) implantation (from prior instrumentation); 3) retrograde venous flow; and 4) arterial or lymphatic dissemination. A review conducted by Mearini et al. pointed out that the communication between the prostatic plexus of Santorini and the deep dorsal vein of the penis is the main reason for the higher incidence of penile secondary tumors from PCa (18). Moreover, they also found that most penile metastases were located in the corpora cavernosa. Subsequently, we also documented that penile shaft and corpora cavernosa were the mostly location of metastases (**Table 1**). Furthermore, one patient in our report received radical penectomy, and the histopathology results of the surgical specimen revealed the tumor emboli in the vein of the penile cavernosa. Therefore, retrograde venous flow was deemed the most common pathway of penile metastasis from the prostate.

Previous reports have shown that the prognosis of metastatic penile cancer is very poor, and few patients have been reported to survive for 2 years or more (19). Moreover, the average survival is reported to be 6 months upon presentation of penile metastases from PCa (20). In our report, the average survival after diagnosing penile metastases was 10 months, which was better than that previously reported. Early diagnosis and treatment were the major reasons. The choice of treatment generally depends on patient clinical status and disease burden, and treatments include chemotherapy, radiotherapy and hormonal therapy. Partial or radical penectomy may be performed for patients with priapism or uncontrollable pain (20). However, for metastatic PCa, the main treatment is androgen deprivation therapy (ADT). After developing to the lethal castration-resistant PCa (CRPC), new hormonal therapy (i.e., abiraterone, enzalutamide) and chemotherapy (i.e., docetaxel) have shown efficacy in improving overall survival for these patients. In addition, local radiotherapy will improve the control of locoregional tumor and relieve the local symptoms (uncontrollable pain), which also improve the prognosis and quality of life. Moreover, ^177^Lu-labeled PSMA radioligand therapy has also shown encouraging results.

PSMA PET/CT may identify asymptomatic penile metastases and has important implications for disease management. In a case report by Mansbridge et al., asymptomatic penile metastases were found by PSMA PET/CT, and then this patient received local palliative radiotherapy (15). Fortunately, after 18 months, this patient was still alive and was being prepared for chemotherapy. Furthermore, Davidson et al. recently reported the characteristics of 24 patients with penile metastases by reviewed ^18^F-FDG and ^68^Ga-PSMA PET/CT records in their institution (21). Subsequently, they pointed that prostate was the most common primary site of penile metastases, and 12 (50%) patients with penile metastases from PCa, which was higher than previous reported. All the penile metastases of these patients were detected by PSMA PET/CT, which was also consistent with our observation. This indicates that the incidence of penile metastasis from PCa may be more frequent than previous knowledge with wide application of PSMA PET/CT. Also, the median survival was 11.5 months in 6 cases with PCa, which was better than previous reports. The reason of this phenomena may be due to an early diagnosis and more effective treatment. However, the initial local symptoms of these patients were not detailed in their study. In our report, a patient with asymptomatic penile metastases was also diagnosed by ^18^F-PSMA PET/CT followed by lesion biopsy and at the stage of castration-sensitive. PSMA PET/CT have a better ability to identify asymptomatic penile metastases, when comparing with conventional imaging [ultrasonography (Case 1), MRI (Case 2)] and provide the accuracy staging of PCa. Based on the accurate staging of PCa, we will perform more effective treatment in advance (i.e., local radiotherapy, chemotherapy, abiraterone and enzalutamide), which will be helpful to improve survival. Despite an aggressive pathological feature (Gleason score 5 + 5 = 10) and low treatment intensity (hormonal therapy), there was no sign of tumor recurrence or progression after 13 months, which was better than previous reports. However, due to the limitation of cases, it is still too soon to assume that an early diagnosis through this technique leads to a survival advantage, but rather to an increase in the time of disease consciousness. PSMA PET/CT is a novel imaging technique, which can not only increase the accuracy of PCa staging, but also help to identify some distant uncommon site metastases (i.e, brain, penis) when comparing to other conventional imaging. Subsequently, it will lead to a more accurate diagnosis and effective treatment for patients. Early detection at the stage of hormone-sensitive PCa and more active treatment may contribute to a survival advantage. However, this conclusion still needs to be confirmed by more studies to rule out the effects of time bias in future.

## Conclusion

Penile metastases from PCa are extremely rare and typically indicate an advanced stage disease and a poor prognosis. PSMA PET/CT may detect more asymptomatic penile metastases in the future. In addition, early diagnosis and treatment can result in survival advantages.

## Data Availability Statement

The original contributions presented in the study are included in the article/supplementary material. Further inquiries can be directed to the corresponding author.

## Ethics Statement

Written informed consent was obtained from the individual(s) for the publication of any potentially identifiable images or data included in this article.

## Author Contributions

JF: data collection, literature search, and manuscript writing. HL: literature searching, pathological specimens reviewing, and manuscript writing. XZ: data collection and manuscript writing. XC: perform biopsy and manuscript editing. XD: PET/CT data collection and manuscript writing. LL: operation performance and manuscript editing. DH: project development and manuscript editing. KW: project development, operation performance, and manuscript editing. All authors contributed to the article and approved the submitted version.

## Funding

This study was supported by the Clinical Application Research Grant of the First Affiliated Hospital of Xi’an Jiaotong University (to LL).

## Conflict of Interest

The authors declare that the research was conducted in the absence of any commercial or financial relationships that could be construed as a potential conflict of interest.
